# Comparable Access, Different Outcomes: Breast Cancer Survival Among Syrian Refugees and Turkish Patients in Türkiye

**DOI:** 10.3390/curroncol33030155

**Published:** 2026-03-08

**Authors:** Ilker Nihat Ökten, Tuba Baydaş, Canan Karan, Oğuzhan Kesen, İbrahim Çil, Fatih Teker

**Affiliations:** 1 Department of Medical Oncology, Göztepe Prof. Dr. Süleyman Yalçın City Hospital, Istanbul 34722, Türkiye; tuba.baydas@gmail.com; 2 Özel Denizli Tekden Hastanesi, Denizli 20010, Türkiye; canankaran@hotmail.com; 3 Oğuzhan Kesen, Rize Recep Tayyip Erdoğan University Hospital, Rize 53020, Türkiye; doc_ozi@hotmail.com; 4 Department of Medical Oncology, SBÜ Ümraniye Research and Training Hospital, Istanbul 34764, Türkiye; dribrahimcil@gmail.com; 5 Özel Liv Hospital, Gaziantep 27080, Türkiye

**Keywords:** breast cancer, Syrian refugees, health equity, migration, survival analysis, universal healthcare

## Abstract

**Background:** Breast cancer outcomes are influenced by tumor biology, stage at diagnosis, and access to timely care. Refugee populations may experience disparities in cancer outcomes despite formal access to healthcare services. Türkiye hosts the largest population of Syrian refugees globally and provides universal access to oncology care, offering a unique context to examine equity in breast cancer outcomes. **Methods:** We performed a retrospective cohort study of female patients diagnosed with invasive breast cancer between 2013 and 2022 at two tertiary oncology centers in Gaziantep, Türkiye. Patients were grouped as Syrian refugees or Turkish citizens based on recorded nationality. Baseline clinicopathologic features and stage at diagnosis were compared between groups. Overall survival (OS) was estimated by the Kaplan–Meier method and compared using log-rank tests. Survival analyses were performed overall and stratified by stage category (I–III vs. IV). Cox proportional hazards regression was used to evaluate the association between ethnicity and OS with adjustment for stage and molecular subtype (and other prespecified covariates as appropriate). Treatment delivery patterns (systemic therapy and radiotherapy) were descriptively compared to evaluate access after entry into care. **Results:** Among 499 patients (150 Syrian refugees; 349 Turkish citizens), Syrian patients were younger at diagnosis and more frequently presented with de novo metastatic disease. In the overall cohort with survival data (*n* = 430), unadjusted OS differed by ethnicity; however, survival differences were attenuated after stratification by stage. In stage I–III disease, OS did not significantly differ between groups, and in stage IV disease, median OS was comparable between ethnicities. In multivariable analysis adjusting for stage and molecular subtype, ethnicity was not independently associated with OS, whereas stage and molecular subtype remained prognostic. Treatment delivery patterns in both the non-metastatic and metastatic settings were broadly similar between groups. **Conclusions:** Within a universal healthcare system, the dominant disparity between Syrian refugees and Turkish citizens was more advanced stage at presentation. After accounting for stage and tumor biology, ethnicity itself was not independently associated with overall survival, suggesting that efforts to reduce outcome gaps should prioritize earlier diagnosis and linkage to care.

## Simple Summary

Breast cancer outcomes can vary between different population groups. Women who have been forced to leave their countries because of war or conflict may face additional challenges when seeking medical care. Türkiye hosts the largest population of Syrian refugees in the world and provides access to cancer treatment through its public healthcare system. In this study, we compared Syrian refugee women with Turkish women who were treated for breast cancer at two cancer centers in southeastern Türkiye. We examined how the disease was diagnosed, how patients were treated, and how long they survived after diagnosis. Syrian women were generally younger when they were diagnosed and were more likely to have cancer that had already spread to other parts of the body. However, once patients entered the healthcare system, both groups received similar cancer treatments. When we considered factors such as the stage of the disease and the biological characteristics of the tumor, refugee status itself was not linked to worse survival. These findings suggest that the main challenge is not unequal treatment after diagnosis but the later stage of disease at the time cancer is detected. Improving early diagnosis and access to screening may help reduce these differences.

## 1. Introduction

Breast cancer is the most frequently diagnosed malignancy among women worldwide and represents a major contributor to global cancer mortality, accounting for more than 2.3 million new cases annually [1]. Despite significant advances in screening and treatment, disparities in breast cancer presentation, tumor biology, and survival persist across population groups, particularly in settings affected by socioeconomic inequality, instability, or forced migration [2].

Since the onset of the Syrian conflict in 2011, more than 6.6 million individuals have been forcibly displaced, with Türkiye hosting the largest population of Syrian refugees under temporary protection status [3,4]. Although Türkiye provides comprehensive and free access to oncology services for Syrian refugees within its public healthcare system, prior research suggests that healthcare accessibility alone does not fully eliminate disparities in cancer outcomes [5]. Structural and social barriers—including language limitations, economic hardship, reduced health literacy, cultural beliefs, transportation difficulties, and challenges in continuity of care—continue to influence cancer diagnosis and treatment among refugee populations [6,7,8].

Evidence from migrant and refugee health research indicates that displaced women are more likely to present with advanced-stage breast cancer and have lower participation in screening programs compared with host populations [9,10]. Delayed initiation of systemic therapy, reduced treatment adherence, and lower rates of guideline-concordant care have also been reported, potentially contributing to poorer outcomes [11,12]. Psychosocial stressors related to war exposure, displacement, and socioeconomic instability may further influence health-seeking behavior and disease trajectory [13,14].

These disparities may arise from a combination of structural barriers and biological differences. In socially and economically disadvantaged populations, breast cancer is often diagnosed at younger ages and may be associated with more aggressive tumor biology, including higher proliferative indices and increased prevalence of HER2-positive and triple-negative subtypes [15,16,17]. Early-onset breast cancer, which is more common in vulnerable populations, has been linked to distinct tumor characteristics and less favorable prognosis [18].

Although data on breast cancer outcomes among Syrian refugee populations are gradually emerging, available evidence remains limited and heterogeneous. Several studies from Türkiye and the Middle East have reported more advanced disease at presentation and inferior survival among Syrian women compared with host populations, even when treated within the same healthcare system [19,20,21]. However, comprehensive analyses that simultaneously evaluate clinicopathologic features, treatment access, and survival across different treatment settings remain scarce.

In this study, we compared clinicopathologic characteristics, treatment access, and survival outcomes between Syrian refugee and Turkish breast cancer patients treated at two tertiary oncology centers in Gaziantep, Türkiye. We aimed to determine whether differences in overall survival persisted after accounting for age, molecular subtype, and treatment setting, and whether access to standard oncologic therapies was comparable between ethnic groups. By stratifying analyses across metastatic, neoadjuvant, and adjuvant treatment contexts, we sought to disentangle biological, clinical, and structural contributors to outcome disparities within a universal healthcare setting.

## 2. Methods

### 2.1. Study Design and Setting

This retrospective, observational cohort study included female patients diagnosed with breast cancer and treated at a tertiary oncology center located in Gaziantep, a province hosting one of the largest Syrian refugee populations in Türkiye, where both refugee and Turkish patients receive cancer care within the same public healthcare system between 2013 and 2022. Given the potential for intercity mobility among refugee populations, inclusion was limited to patients who continued treatment and follow-up at the same center to ensure accurate outcome assessment. Clinical, pathological, and treatment-related data were extracted from electronic medical records. Patients were categorized as Syrian refugees or Turkish citizens according to nationality information recorded in the hospital registration system. All patients were managed within the same healthcare system under standardized national oncologic treatment guidelines. As this was a retrospective observational study including all eligible patients treated during the study period, no formal sample size calculation was performed.

### 2.2. Eligibility Criteria

Patients were eligible if they:

1. Had pathologically confirmed invasive breast cancer;
2. Received at least one modality of oncologic treatment (surgery, chemotherapy, radiotherapy, endocrine therapy, or targeted therapy);
3. Had sufficient baseline demographic and clinical data;
4. Had documented vital status and follow-up time (or date of last contact) for survival analyses.

Patients with missing essential diagnostic or treatment data were excluded.

### 2.3. Data Collection

The following variables were recorded:

Sociodemographic data: age at diagnosis, menopausal status, parity, ethnicity.

Clinical characteristics: stage at presentation, presence of metastatic disease, tumor size, nodal status, molecular subtype, Ki-67 proliferation index.

Stage at diagnosis (AJCC I–IV) was recorded at initial presentation based on pathological and radiological staging documentation available in the electronic medical record; when insufficient documentation was present, the stage was coded as missing.

Pathological features: histologic type, ER/PR/HER2 status, HER2 FISH results, tumor grade.

Imaging metrics: PET-CT positivity, SUVmax values at diagnosis.

Treatment modalities: neoadjuvant therapy, type of breast surgery (mastectomy vs. breast-conserving surgery), axillary surgery (SLNB vs. ALND), adjuvant therapy, first- to fourth-line systemic treatments in metastatic patients.

Outcome measures: overall survival (OS), progression-free survival (PFS), pathological response to neoadjuvant therapy, and vital status.

### 2.4. Definitions

Molecular subtypes were classified as luminal A, luminal B, HER2-positive, or triple-negative according to standard immunohistochemical criteria.

Overall survival (OS) was defined as the interval from diagnosis to death from any cause or last follow-up.

Progression-free survival (PFS) (metastatic cohort) was defined as time from first-line therapy initiation to documented progression or death.

Pathological complete response (pCR) was defined as the absence of invasive tumor in breast and axilla after neoadjuvant therapy.

### 2.5. Statistical Analysis

Descriptive statistics were used to summarize baseline sociodemographic, clinicopathologic, and treatment-related variables. Continuous variables were assessed for distributional assumptions and are presented as mean ± standard deviation or median [interquartile range], as appropriate. Categorical variables are presented as counts and percentages.

Comparisons between Syrian refugees and Turkish citizens were performed using Student’s *t*-test for normally distributed continuous variables and the Mann–Whitney U test for non-normally distributed variables. Categorical variables were compared using the Pearson χ^2^ test; Fisher’s exact test was applied when expected cell counts were <5. Stage at diagnosis (AJCC I–IV) was compared between ethnic groups using the χ^2^ test. In subgroup analyses restricted to non-metastatic patients, stage distribution (I–III) was similarly compared between ethnic groups.

Overall survival (OS) was analyzed using the Kaplan–Meier method, with group differences assessed by the log-rank (Mantel–Cox) test. Median follow-up duration was estimated using the reverse Kaplan–Meier method. To account for imbalance in stage at presentation, survival analyses were also stratified by stage category (Stage I–III vs. Stage IV), and additional comparisons were performed across combined stage–ethnicity groups.

The association between ethnicity and OS was evaluated using Cox proportional hazards regression. Univariate Cox models were initially fitted, followed by multivariable models adjusting for clinically relevant covariates (stage category and molecular subtype). Covariates were retained based on clinical relevance and feasibility given the number of observed events. Analyses were performed using a complete-case approach for variables included in each model.

Analyses were conducted using a complete-case approach. Cases with missing values for specific variables were excluded from the corresponding analyses, and no imputation procedures were applied.

All tests were two-sided, and a *p* value < 0.05 was considered statistically significant. Statistical analyses were conducted using IBM SPSS Statistics for Windows, version 26.0 (IBM Corp., Armonk, NY, USA).

### 2.6. Use of Artificial Intelligence Tools

Artificial intelligence-based tools (ChatGPT 5.2, OpenAI) were used to assist with statistical methodology clarification and language editing during manuscript preparation. All analyses were designed, conducted, verified, and interpreted by the authors, who take full responsibility for the integrity and accuracy of the data and results.

## 3. Results

A total of 499 breast cancer patients were included in the analysis, comprising 150 Syrian refugees and 349 Turkish citizens. Syrian patients were significantly younger at diagnosis than Turkish patients (median age 45 [IQR 16] vs. 47 [IQR 17] years, *p* = 0.002) and had a higher parity (median 5 [IQR 3] vs. 4 [IQR 2], *p* = 0.008). The Ki-67 proliferation index was significantly higher in Syrian patients (median 20% [IQR 20] vs. 13.5% [IQR 15], *p* = 0.027), whereas age at menopause did not differ between groups.

Stage at diagnosis differed significantly between ethnic groups (*p* < 0.001). Syrian refugees were substantially more likely to present with de novo metastatic disease compared with Turkish citizens (46.0% vs. 24.9%). Conversely, early-stage disease (stage I–II) was more common among Turkish citizens. Stage information was available for 493 patients (98.8%) (Table 1).

Initial treatment approach differed significantly according to ethnicity (*p* < 0.001), with Syrian patients more frequently receiving treatment in the metastatic setting and Turkish patients more commonly treated with neoadjuvant therapy. Although a higher proportion of non-luminal subtypes was observed among Syrian patients, this difference did not reach statistical significance. Histologic subtype, estrogen receptor status, HER2 expression, tumor grade, molecular subtype distribution, laterality, family history, and menopausal status were comparable between groups. Progesterone receptor negativity was more frequent among Syrian patients compared with Turkish patients (40.8% vs. 32.1%, *p* = 0.040). At last follow-up, a higher proportion of deaths was observed among Syrian patients with a difference at the threshold of statistical significance (47.6% vs. 39.1%, *p* = 0.050) (Table 1).

Overall Survival According to Ethnicity

**Figure 1:**
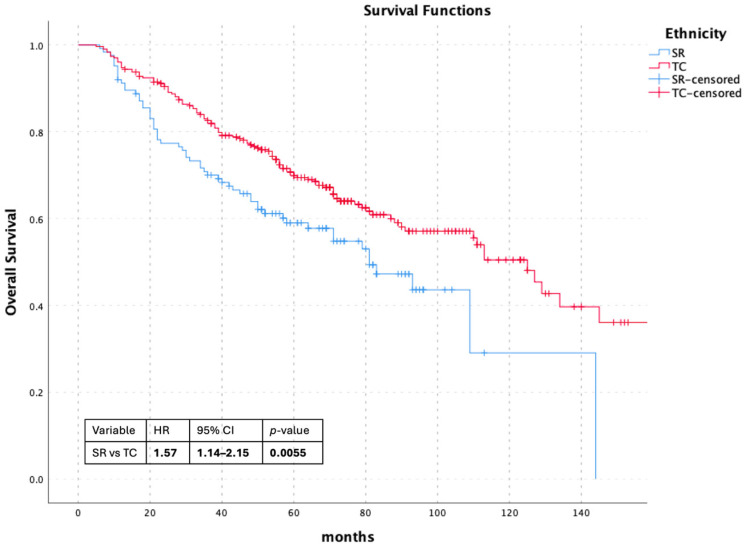
Overall Survival According to Ethnicity.

In the overall cohort with available survival data (*n* = 430), univariate Cox proportional hazards analysis demonstrated that Syrian refugee status was associated with significantly worse overall survival compared with Turkish citizens (HR 1.57, 95% CI 1.14–2.15; *p* = 0.006). Kaplan–Meier analysis confirmed this difference (log-rank *p* = 0.005) (Figure 1).

Stage-Stratified Survival Analysis

Given the imbalance in stage distribution at diagnosis between ethnic groups, stage-stratified survival analyses were performed using a four-group categorization (Stage I–III Turkish, Stage I–III Syrian, Stage IV Turkish, and Stage IV Syrian).

**Figure 2:**
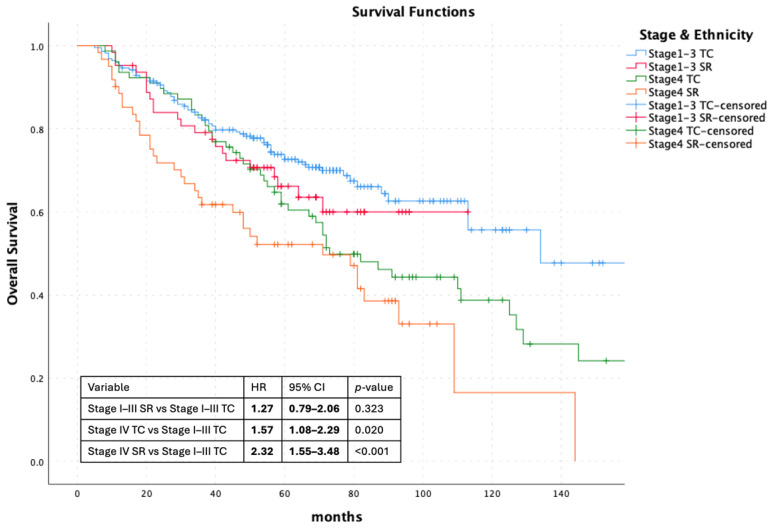
Overall Survival According to Stage & Ethnicity.

Overall survival differed significantly across these four groups (log-rank χ^2^ = 18.7, *p* < 0.001) (Figure 2).

When Stage I–III patients were analyzed separately, no statistically significant difference in survival was observed between Syrian and Turkish patients (HR 1.27, 95% CI 0.79–2.06; *p* = 0.323). In contrast, Stage IV disease was associated with significantly worse survival compared with Stage I–III Turkish patients (reference group), irrespective of ethnicity (Stage IV Turkish: HR 1.57, 95% CI 1.08–2.29; *p* = 0.020; Stage IV Syrian: HR 2.32, 95% CI 1.55–3.48; *p* < 0.001).

Summary of median survival and follow-up.

To summarize survival outcomes by stage category and ethnicity, median overall survival (OS) estimates and the number of deaths are presented in Table 2. In the overall cohort, OS differed significantly between Syrian refugees and Turkish citizens (log-rank *p* = 0.005). However, when analyses were stratified by stage, no statistically significant survival difference was observed between ethnic groups among patients with Stage I–III disease (log-rank *p* = 0.342) or Stage IV disease (log-rank *p* = 0.982).

These findings suggest that the observed survival difference in the overall cohort may be influenced by stage distribution at diagnosis. Given the observed imbalance in stage distribution and the attenuation of survival differences after stage stratification, we next performed multivariable Cox proportional hazards analysis to evaluate whether ethnicity independently predicted overall survival.

In univariate Cox regression analysis, Syrian ethnicity was associated with significantly worse overall survival compared with Turkish patients (HR 1.57, 95% CI 1.14–2.15; *p* = 0.006). Advanced stage at diagnosis (Stage IV vs. Stage I–III) was also strongly associated with inferior survival (HR 1.75, 95% CI 1.29–2.36; *p* < 0.001). Molecular subtype was significantly associated with prognosis, with Luminal B (HR 2.29, 95% CI 1.37–3.83; *p* = 0.002), HER2-positive (HR 1.96, 95% CI 1.18–3.24; *p* = 0.009), and triple-negative tumors (HR 2.76, 95% CI 1.46–5.22; *p* = 0.002) demonstrating higher hazards of death compared with Luminal A disease. Age at diagnosis was not significantly associated with overall survival (HR 1.01 per year increase, 95% CI 0.99–1.02; *p* = 0.482).

In multivariable analysis adjusting for stage and molecular subtype, ethnicity was no longer independently associated with overall survival (adjusted HR 1.15, 95% CI 0.80–1.65; *p* = 0.445). Advanced stage remained an independent predictor of worse survival (adjusted HR 1.66, 95% CI 1.19–2.32; *p* = 0.003). Molecular subtype also remained independently associated with prognosis, with Luminal B (adjusted HR 2.14, 95% CI 1.28–3.60; *p* = 0.004), HER2-positive (adjusted HR 1.74, 95% CI 1.05–2.90; *p* = 0.032), and triple-negative tumors (adjusted HR 2.79, 95% CI 1.47–5.30; *p* = 0.002) showing significantly higher mortality risk compared with Luminal A tumors (Table 3).

To better characterize differences according to disease stage, patients were analyzed in two main cohorts: those presenting with metastatic disease (stage IV) and those with non-metastatic disease (stage I–III). The non-metastatic cohort was further stratified according to treatment strategy into patients receiving neoadjuvant systemic therapy and those treated in the adjuvant setting.

### 3.1. Stage IV Disease (Metastatic Cohort)

Among patients with metastatic breast cancer and known ethnicity, 69 were Syrian refugees and 87 were Turkish citizens. Syrian refugees tended to be younger at metastatic diagnosis compared with Turkish citizens, although this difference did not reach statistical significance (47.3 ± 13.2 vs. 52.7 ± 12.8 years, *p* = 0.056). Pre-treatment tumor diameter and PET SUVmax at diagnosis were comparable between the two groups (*p* = 0.107 and *p* = 0.458, respectively).

Tumor grade distribution did not differ by ethnicity (*p* = 0.821). Molecular subtype distribution was also similar between groups when analyzed using the four-category classification (*p* = 0.314). Using a binary molecular classification, there was a trend toward a higher proportion of non-luminal tumors among Syrian refugees compared with Turkish citizens (61.4% vs. 45.2%), although this did not reach statistical significance (*p* = 0.067).

The use of PET imaging at diagnosis was comparable between the two groups (*p* = 1.000). Patterns of metastatic involvement, including lung, liver, bone, brain, lymph node, and other metastatic sites, were similar across ethnicities, with no statistically significant differences observed (Table 4).

**Table 4:** Baseline characteristics of the metastatic cohort.

Variable	Syrian Refugees	Turkish Citizens	*p* Value
**Age at metastatic diagnosis, years**	47.26 ± 13.23	52.74 ± 12.80	0.056
**Pre-treatment tumor diameter, mm**	47.55 ± 27.59	34.85 ± 14.05	0.107
**PET SUVmax at diagnosis**	12.00 ± 7.10	11.12 ± 7.17	0.458
**Tumor grade**	43	50	0.821
Grade 1	1 (2.3%)	1 (2.0%)	
Grade 2	25 (58.1%)	26 (52.0%)	
Grade 3	17 (39.5%)	23 (46.0%)	
**Molecular subtype (4-group)**	57	73	0.314
Luminal A	6 (10.5%)	13 (17.8%)	
Luminal B	16 (28.1%)	27 (37.0%)	
HER2-enriched	29 (50.9%)	27 (37.0%)	
TNBC	6 (10.5%)	6 (8.2%)	
**Molecular subtype (binary)**	57	73	0.067
Luminal type	22 (38.6%)	40 (54.8%)	
Non-luminal (HER2/TNBC)	35 (61.4%)	33 (45.2%)	
**PET performed at diagnosis**	55	62	1.000
Yes	31 (56.4%)	35 (56.5%)	
No	24 (43.6%)	27 (43.5%)	
**Sites of metastasis**			
Lung metastasis	25 (47.2%)	29 (47.5%)	1.000
Liver metastasis	18 (39.1%)	26 (38.8%)	1.000
Bone metastasis	44 (74.6%)	60 (77.9%)	0.801
Brain metastasis	6 (15.0%)	17 (30.4%)	0.135
Lymph node metastasis	26 (54.2%)	33 (55.0%)	1.000
Other metastasis	3 (7.9%)	4 (7.5%)	1.000

Footnote: Similarly, second-line systemic therapy distributions were comparable between the two groups (*p* = 0.303). The use of palliative radiotherapy was numerically lower among Syrian refugees compared with Turkish citizens; however, this difference did not reach statistical significance (*p* = 0.120). Overall, these findings suggest similar access to systemic and local treatment modalities between Syrian refugee and Turkish patients in the metastatic setting (Table 5).

**Table 5:** Access to systemic therapy and radiotherapy in metastatic breast cancer patients according to ethnicity.

Treatment Modality	Syrian Refugees, *n* (%)	Turkish Citizens, *n* (%)	*p* Value
**First-line metastatic treatment**			0.219
Aromatase inhibitor/Tamoxifen	14 (20.6)	26 (31.7)	
Aromatase inhibitor + CDK4/6 inhibitor	11 (16.2)	18 (22.0)	
Chemotherapy	16 (23.5)	17 (20.7)	
Chemotherapy + trastuzumab + pertuzumab	18 (26.5)	11 (13.4)	
Chemotherapy + trastuzumab	9 (13.2)	10 (12.2)	
**Second-line metastatic treatment**			0.303
Aromatase inhibitor/Fulvestrant	9 (20.9)	13 (24.5)	
Aromatase inhibitor/Fulvestrant + CDK4/6 inhibitor	6 (14.0)	14 (26.4)	
Chemotherapy	13 (30.2)	8 (15.1)	
Chemotherapy + trastuzumab	6 (14.0)	5 (9.4)	
Lapatinib or T-DM1	9 (20.9)	13 (24.5)	
**Radiotherapy**			0.120
Palliative radiotherapy	17 (40.5)	32 (56.1)	

Footnote: Data are presented as number (%). Comparisons between groups were performed using the Pearson chi-square test. Percentages are calculated among patients who received the respective line of treatment. CDK4/6, cyclin-dependent kinase 4/6; T-DM1, ado-trastuzumab emtansine.

### 3.2. Stage I–III Disease (Non-Metastatic Cohort)

#### 3.2.1. Neoadjuvant-Treated Patients

In the neoadjuvant-treated subgroup, baseline tumor characteristics, surgical approaches, axillary management, molecular subtype distribution, and pathologic response rates were comparable between Syrian refugee and Turkish patients. Clinical stage distribution at diagnosis was also similar between the two groups, with most patients presenting with stage II–III disease (stage I: 2.2% vs. 1.7%, stage II: 60.9% vs. 58.9%, stage III: 37.0% vs. 39.4% for Syrian refugees and Turkish citizens, respectively; *p* = 0.934). Pre-treatment and residual tumor diameters did not differ significantly between ethnic groups.

Importantly, access to neoadjuvant systemic therapy—including anthracycline-based chemotherapy, taxane-containing regimens, and anti-HER2 therapy—was similar across ethnicities, indicating equitable delivery of standard-of-care treatment in this cohort (Table 6).

Among patients with HER2-positive breast cancer treated in the neoadjuvant setting, the distribution of anti-HER2 treatment regimens was compared according to ethnicity. Dual HER2 blockade with taxane, trastuzumab, and pertuzumab was numerically more frequent among Turkish citizens compared with Syrian refugees; however, this difference did not reach statistical significance (Fisher’s exact test, *p* = 0.261). These findings indicate no statistically significant disparity in access to contemporary neoadjuvant anti-HER2 therapy between the two ethnic groups (Table 7).

#### 3.2.2. Adjuvant-Treated Patients

Baseline clinicopathologic characteristics were largely comparable between Syrian refugees and Turkish citizens in the adjuvant cohort. Although a higher proportion of Syrian patients were classified as stage III disease (41.2% vs. 19.5%, *p* = 0.011), the absolute numbers were small (14 vs. 15 patients), and this difference should be interpreted cautiously. Surgical approach and molecular subtype distribution were otherwise similar between the groups (Table 8).

## 4. Discussion

Our study provides a comprehensive comparative analysis of breast cancer presentation, treatment patterns, and survival outcomes between Syrian refugees and Turkish citizens treated within the same universal healthcare system. The results highlight a critical dichotomy: while Syrian refugees present with significantly more advanced disease and at a younger age, their survival outcomes are comparable to Turkish citizens when adjusted for disease stage and biology.

### 4.1. Demographic and Clinicopathologic Differences

A key finding of our study is the significantly younger age at diagnosis among Syrian refugees (median 45 vs. 47 years). This aligns with prior reports from Türkiye and the Middle East, which consistently describe a younger age of onset in displaced populations [10,19,20]. This demographic difference has profound implications for screening; while national screening programs in Türkiye typically target women over 40, migration-related disruptions may prevent younger refugee women from engaging with these services [7]. Additionally, we observed a significantly higher Ki-67 proliferation index and a trend toward non-luminal subtypes in the refugee cohort. While this may reflect the “healthy migrant effect” in reverse—where only the most symptomatic cases present to tertiary centers—it suggests a need for high clinical suspicion even in younger refugee patients [12].

### 4.2. Psychosocial and Structural Contributors

Beyond biological factors, psychosocial and structural stressors related to forced displacement may further influence cancer outcomes. Chronic stress, economic insecurity, and prior trauma have been associated with altered health behaviors and reduced adherence to long-term treatments [13,14]. Furthermore, experimental models have demonstrated that chronic stress can dysregulate neuroendocrine and immunological pathways—specifically via catecholamine and glucocorticoid signaling—which may promote tumor metastasis and resistance to therapy [22]. Compounded by the psychological burden of war exposure, these stressors create a “biological environment” that may accelerate disease progression. Although we restricted our cohort to patients who continued treatment and follow-up at the same center to minimize loss to follow-up, residual instability in living conditions may still have affected outcomes among refugee patients [5].

### 4.3. Survival Analysis and the Burden of Late Presentation

The most striking disparity in our cohort was stage at presentation. Nearly half of Syrian refugees were diagnosed with stage IV disease, compared with approximately one-quarter of Turkish citizens. This substantial imbalance in stage distribution likely explains much of the observed survival difference in unadjusted analyses [19,20,21].

Importantly, once stage and molecular subtype were incorporated into multivariable models, ethnicity was no longer independently associated with overall survival. These findings reinforce that delayed presentation, rather than differential treatment access, is the principal driver of outcome disparities in this setting [9,12].

### 4.4. Health Equity Implications

From a health equity perspective, our findings emphasize the distinction between *horizontal equity* (equal access to healthcare services) and *vertical equity* (care delivery tailored to differing needs). Our data indicate that Türkiye has largely achieved horizontal equity; once diagnosed, Syrian refugees received neoadjuvant and metastatic treatments at rates comparable to Turkish citizens.

However, the persistent gap in stage at diagnosis suggests a failure in vertical equity. Even within a universal healthcare system, refugee populations face “health literacy” barriers—defined not just as the ability to read, but the competency to access, understand, and apply health information to make decisions [23]. Syrian refugees may require additional support mechanisms, such as culturally adapted health education, patient navigation services, and interpreter availability, to bridge the gap between “access to care” and “early diagnosis” [7].

### 4.5. Limitations and Strengths

This study has several limitations that should be acknowledged. Its retrospective design limits causal inference, and some clinically relevant variables—such as time from symptom onset to diagnosis, socioeconomic status, and treatment adherence—were not uniformly available. Overall survival was used as the primary endpoint, which may be influenced by non-cancer-related mortality. Additionally, restricting inclusion to patients who continued treatment at the same center may have introduced selection bias toward more stable refugee patients, potentially underestimating the true magnitude of disparities. Nevertheless, this approach was necessary to ensure reliable longitudinal follow-up in a highly mobile population.

Despite these limitations, our study has notable strengths. It was conducted in a real-world setting within a unified public healthcare system, included a relatively large refugee cohort, and applied a setting-stratified analytical approach that allowed nuanced assessment of access and outcomes across different treatment contexts. To our knowledge, this is one of the most comprehensive comparisons of Syrian refugee and host-country breast cancer patients in Türkiye to date.

## 5. Conclusions

In conclusion, while Syrian refugees present with more advanced breast cancer and at a younger age than Turkish citizens, the lack of independent survival disparity in multivariate analysis confirms that the Turkish healthcare system provides equitable oncologic treatment. Future efforts must move beyond treatment access to address the structural and psychosocial barriers that delay diagnosis, employing “vertical equity” strategies to ensure refugees can access care at a curable stage.

## Figures and Tables

**Table 1:** Clinicopathologic and sociodemographic characteristics according to ethnicity.

Variables	Syrian Refugees (*n* = 150)	Turkish Citizens (*n* = 349)	*p* Value
**Age, years**	45 [16]	47 [17]	**0.002**
**Age at menopause, years**	50 ± 5.56	49 ± 4.64	0.262
**Parity**	5 [3]	4 [2]	**0.008**
**Ki-67, %**	20 [20]	13.5 [15]	**0.027**
**Stage at Diagnosis**			**<0.001**
Stage I	4 (2.7)	27 (7.7)	
Stage II	45 (30.0)	144 (41.3)	
Stage III	31 (20.7)	86 (24.6)	
Stage IV	69 (46.0)	87 (24.9)	
Missing	1 (0.7)	5 (1.4)	
**Initial treatment approach**			**<0.001**
Metastatic treatment	69 (46.0)	87 (24.9)	
Neoadjuvant therapy	46 (30.7)	184 (52.7)	
Adjuvant therapy	35 (23.3)	78 (22.3)	
**Histology**			0.548
Invasive ductal carcinoma	131 (90.3)	318 (94.1)	
Invasive lobular carcinoma	11 (7.7)	16 (4.7)	
Other	3 (2.1)	4 (1.2)	
**Estrogen receptor (ER)**			0.307
Negative	42 (28.6)	82 (23.8)	
Positive	105 (71.4)	263 (76.2)	
**Progesterone receptor (PR)**			**0.040**
Negative	60 (40.8)	111 (32.1)	
Positive	87 (59.2)	235 (67.9)	
**HER2 (IHC)**			0.860
0/1+/2+	95 (66.5)	237 (69.4)	
3+	48 (33.6)	105 (30.7)	
**Tumor grade**			0.953
Grade 1	4 (5.0)	12 (6.2)	
Grade 2	44 (55.0)	105 (54.4)	
Grade 3	32 (40.0)	76 (39.4)	
**Molecular subtype**			0.400
Luminal A	29 (21.8)	78 (24.3)	
Luminal B	33 (24.8)	99 (30.8)	
HER2-enriched	54 (40.6)	112 (34.9)	
TNBC	17 (12.8)	32 (10.0)	
**Molecular subtype (binary)**			0.090
Luminal-type	62 (46.6)	177 (55.1)	
HER2/TNBC	71 (53.4)	144 (44.9)	
**Laterality**			0.808
Right	78 (52.3)	173 (50.3)	
Left	68 (45.6)	166 (48.3)	
Bilateral	3 (2.0)	5 (1.5)	
**Family history**			0.269
None	56 (65.9)	83 (53.2)	
First-degree breast/ovarian cancer	9 (10.6)	19 (12.2)	
First-degree other cancers	8 (9.4)	24 (15.4)	
Other relatives	12 (14.1)	30 (19.2)	
**Menopausal status**			0.679
Premenopausal	56 (65.1)	98 (61.2)	
Postmenopausal	30 (34.9)	62 (38.8)	
**Vital status at last follow-up**			**0.050**
Deceased	69 (47.6)	132 (39.1)	
Alive	76 (52.4)	206 (60.9)	

**Footnote:** Data are presented as mean ± standard deviation, median [interquartile range], or number (%), as appropriate. Continuous variables were compared using Student’s *t*-test or Mann–Whitney U test, and categorical variables using the χ^2^ or Fisher’s exact test. *p*-values refer to overall comparisons across categories. Molecular subtype (binary) represents a simplified biological grouping used for descriptive purposes.

**Table 2:** Overall survival and follow-up duration according to stage group and ethnicity.

Stage Group	Ethnicity	*N*	Deaths *n* (%)	Median Follow-Up (Months, 95% CI)	Median OS (Months, 95% CI)	Log-Rank *p*
All-comers	Syrian	125	59 (47.2%)	74 (66.0–79.0)	81 (59.8–102.1)	**0.005**
	Turkish	305	119 (39.0%)	81 (68.0–91.0)	125 (110.9–139.0)	
Stage I–III	Syrian	63	22 (34.9%)	69 (60.7–77.3)	NR	0.342
	Turkish	224	70 (31.3%)	72 (70.4–73.6)	134 (118.5–149.5)	
Stage IV	Syrian	61	36 (59.0%)	90 (80.3–99.7)	47 (24.0–81.0)	0.982
	Turkish	78	49 (62.8%)	105 (87.5–122.5)	41 (29.0–58.0)	

Table 2 Footnote: Data are presented as *n*, deaths *n* (%), and median OS (months, 95% CI) estimated by the Kaplan–Meier method. Median follow-up was calculated using the reverse Kaplan–Meier method. Survival curves were compared using the log-rank (Mantel–Cox) test; *p*-values correspond to comparisons within each stage category (All-comers, Stage I–III, Stage IV). NR indicates median OS not reached during follow-up.

**Table 3:** Univariate and Multivariate Cox Proportional Hazards Analyses for Overall Survival.

Variable	Category/Unit	Univariate HR (95% CI)	*p*	Multivariate HR (95% CI)	*p*
**Ethnicity**	Syrian vs. Turkish (ref)	1.567 (1.140–2.151)	**0.006**	1.150 (0.804–1.645)	0.445
**Stage**	Stage IV vs. I–III (ref)	1.747 (1.294–2.360)	**<0.001**	1.661 (1.189–2.320)	**0.003**
**Molecular subtype**	Luminal A (ref)	1.000	—	1.000	—
	Luminal B vs. Luminal A	2.287 (1.365–3.833)	**0.002**	2.142 (1.276–3.596)	**0.004**
	HER2 vs. Luminal A	1.958 (1.183–3.243)	**0.009**	1.744 (1.048–2.902)	**0.032**
	TNBC vs. Luminal A	2.756 (1.455–5.219)	**0.002**	2.789 (1.468–5.300)	**0.002**
**Age at diagnosis**	Per year increase	1.005 (0.992–1.018)	0.482	—	—

**Table Footnote:** Hazard ratios (HRs) greater than 1 indicate increased risk of death compared with the reference category. Luminal A was used as the reference category for molecular subtype. Age was not retained in the final multivariable model due to lack of statistical significance and minimal effect size in univariate analysis. The multivariable model included 384 patients with 153 events; univariate analyses were performed using available-case data for each variable.

**Table 6:** Baseline clinicopathologic characteristics and treatment-related features of neoadjuvant breast cancer patients according to ethnicity.

Variable	Syrian Refugees	Turkish Citizens	*p* Value
**Stage at Presentation**	46	180	
Stage 1	1 (2.2%)	3 (1.7%)	
Stage 2	28 (60.9%)	106 (58.9%)	0.934
Stage 3	17 (37%)	71 (39.4%)	
**Tumor characteristics**			
Pre-treatment tumor diameter, mm (mean ± SD)	36.7 ± 18.8 (*n* = 40)	32.8 ± 17.4 (*n* = 175)	0.209
Residual tumor diameter, mm (mean ± SD)	37.1 ± 30.0 (*n* = 23)	29.2 ± 23.5 (*n* = 108)	0.167
**Pre-treatment lymph node status**	40	148	0.617
Positive	33 (82.5%)	129 (87.2%)	
Negative	7 (17.5%)	19 (12.8%)	
**Breast surgery type**	44	167	0.221
Breast-conserving surgery	6 (13.6%)	12 (7.2%)	
Mastectomy	38 (86.4%)	155 (92.8%)	
**Axillary surgery**	44	167	0.359
Sentinel lymph node biopsy (SLNB)	5 (11.4%)	12 (7.2%)	
Axillary lymph node dissection (ALND)	39 (88.6%)	155 (92.8%)	
**Molecular subtype**	41	170	0.459
Luminal	24 (58.5%)	86 (50.6%)	
Non-luminal (HER2/TNBC)	17 (41.5%)	84 (49.4%)	
**Pathologic response to neoadjuvant therapy**	39	164	0.638
No response	17 (43.6%)	72 (43.9%)	
Partial response	6 (15.4%)	35 (21.3%)	
Complete response	16 (41.0%)	57 (34.8%)	

**Table 7:** Neoadjuvant anti-HER2 treatment patterns according to ethnicity in HER2-positive patients.

Neoadjuvant Anti-HER2 Regimen	Syrian Refugees (*n* = 11)	Turkish Citizens (*n* = 46)	*p* Value
Taxane + trastuzumab	10 (90.9%)	33 (71.7%)	0.261
Taxane + trastuzumab + pertuzumab	1 (9.1%)	13 (28.3%)	

Footnote: Percentages are calculated within ethnicity groups. Fisher’s exact test (two-sided) was used due to small expected cell counts.

**Table 8:** Baseline clinicopathologic characteristics and treatment-related features of adjuvant breast cancer patients according to ethnicity.

Variable	Syrian Refugees	Turkish Citizens	*p* Value
**Stage at Presentation**	34	77	
Stage 1	3 (8.8%)	24 (31.2%)	
Stage 2	17 (50%)	38 (49.4%)	**0.011**
Stage 3	14 (41.2%)	15 (19.5%)	
**Tumor characteristics**			
Pre-treatment tumor diameter, mm (mean ± SD)	25.9 ± 14.8	22.7 ± 15.3	0.351
PET SUVmax at diagnosis (mean ± SD)	5.07 ± 2.78	8.49 ± 18.48	0.512
**Surgical characteristics**			
Breast surgery			0.246
Breast-conserving surgery	23 (82.1%)	49 (68.1%)	
Mastectomy	5 (17.9%)	23 (31.9%)	
Axillary surgery			1.000
Sentinel lymph node biopsy (SLNB)	19 (67.9%)	48 (66.7%)	
Axillary lymph node dissection (ALND)	9 (32.1%)	24 (33.3%)	
**Molecular subtype**			
Luminal A	13 (36.1%)	31 (39.7%)	0.174
Luminal B	4 (11.1%)	20 (25.6%)	
HER2-enriched	13 (36.1%)	20 (25.6%)	
Triple-negative	6 (16.7%)	7 (9.0%)	

## Data Availability

The datasets used and/or analyzed during the current study are available from the corresponding author.
